# Supplementary material for: Causal interplay between lactose intolerance and gut microbiota: a combined bidirectional Mendelian randomization and in vivo validation study
Source: Front Nutr. 2026 Jun 1;13:1803337. doi: 10.3389/fnut.2026.1803337 (PMC13265576; doi:10.3389/fnut.2026.1803337)
Supplement: Supplementary file 1 [file Data_Sheet_1.zip › supplementary materials/Forward/leave one out cross validation/ebi-a-GCST90027596.finngen_R12_E4_LACTONAS.pdf]

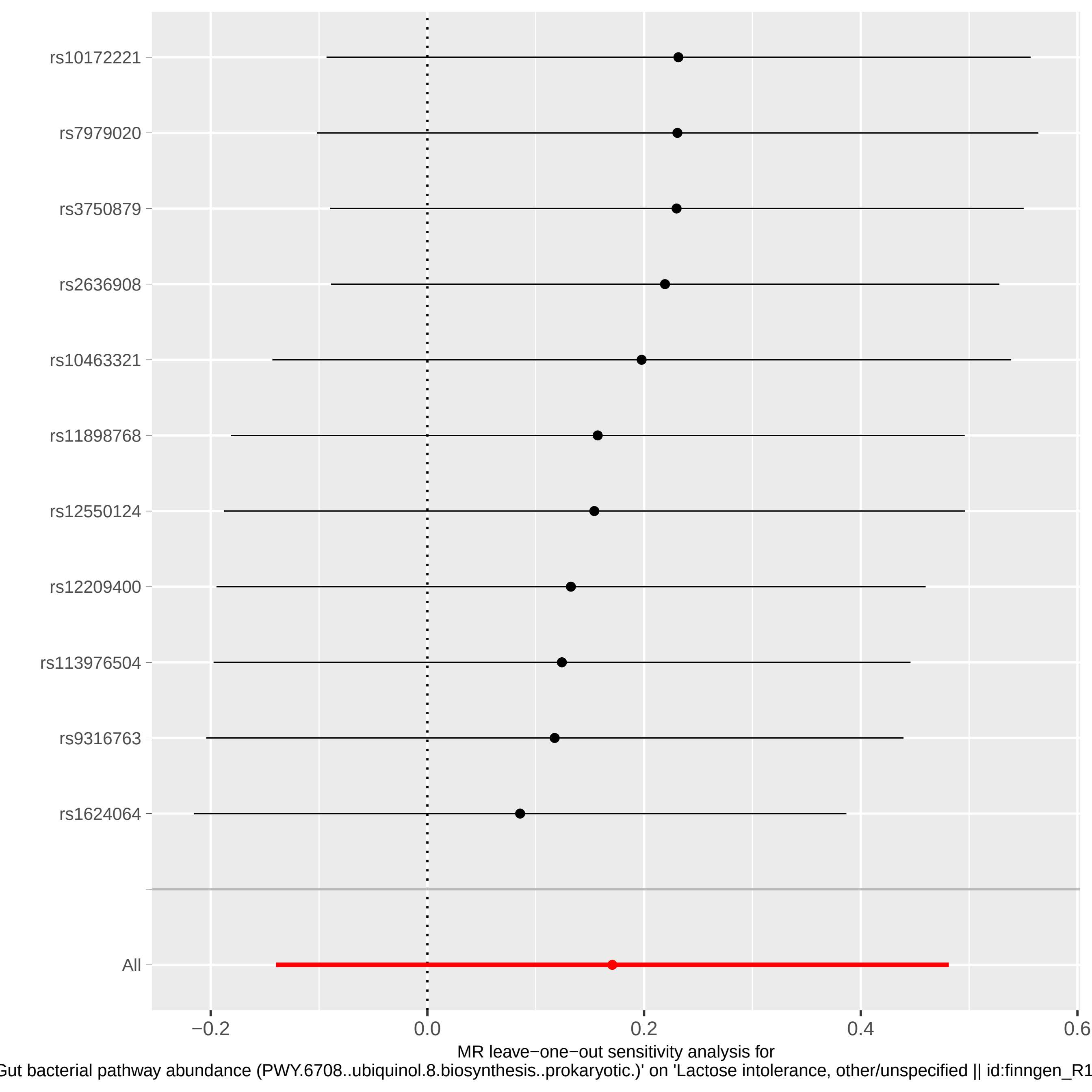
rs10172221
rs7979020
rs3750879
rs2636908
rs10463321
rs11898768
rs12550124
rs12209400
rs113976504
rs9316763
rs1624064
All
−0.2
0.0
0.2
0.4
0.6
MR leave−one−out sensitivity analysis for
Gut bacterial pathway abundance (PWY.6708..ubiquinol.8.biosynthesis..prokaryotic.)' on 'Lactose intolerance, other/unspecified || id:finngen_R1
